# Predicting Medical Students’ Current Attitudes Toward Psychiatry, Interest in Psychiatry, and Estimated Likelihood of Working in Psychiatry: A Cross-Sectional Study in Four European Countries

**DOI:** 10.3389/fpsyt.2018.00049

**Published:** 2018-03-12

**Authors:** Ingeborg Warnke, Alex Gamma, Maria Buadze, Roman Schleifer, Carlos Canela, Bernd Strebel, Tamás Tényi, Wulf Rössler, Nicolas Rüsch, Michael Liebrenz

**Affiliations:** ^1^Department of Forensic Psychiatry, Institute of Forensic Medicine, University of Bern, Bern, Switzerland; ^2^Department of Psychosomatic Medicine, Protestant Hospital Hagen-Haspe, Hagen, Germany; ^3^Department of Psychiatry and Psychotherapy, University of Pécs, Pécs, Hungary; ^4^Department of Psychiatry, Psychotherapy and Psychosomatics, Psychiatric Hospital, University of Zurich, Zurich, Switzerland; ^5^Laboratory of Neuroscience (LIM 27), Institute of Psychiatry, University of São Paulo, São Paulo, Brazil; ^6^Department of Psychiatry and Psychotherapy, Charité University of Medicine, Berlin, Germany; ^7^Department of Psychiatry II, University of Ulm and Bezirkskrankenhaus Günzburg, Ulm, Germany

**Keywords:** attitudes toward psychiatry, interest in psychiatry, professional preference, multivariable modeling, curriculum-related experience, gender, medical school, study year

## Abstract

Psychiatry as a medical discipline is becoming increasingly important due to the high and increasing worldwide burden associated with mental disorders. Surprisingly, however, there is a lack of young academics choosing psychiatry as a career. Previous evidence on medical students’ perspectives is abundant but has methodological shortcomings. Therefore, by attempting to avoid previous shortcomings, we aimed to contribute to a better understanding of the predictors of the following three outcome variables: current medical students’ attitudes toward psychiatry, interest in psychiatry, and estimated likelihood of working in psychiatry. The sample consisted of *N* = 1,356 medical students at 45 medical schools in Germany and Austria as well as regions of Switzerland and Hungary with a German language curriculum. We used snowball sampling *via* Facebook with a link to an online questionnaire as recruitment procedure. Snowball sampling is based on referrals made among people. This questionnaire included a German version of the Attitudes Toward Psychiatry Scale (ATP-30-G) and further variables related to outcomes and potential predictors in terms of sociodemography (e.g., gender) or medical training (e.g., curriculum-related experience with psychiatry). Data were analyzed by linear mixed models and further regression models. On average, students had a positive attitude to and high general interest in, but low professional preference for, psychiatry. A neutral attitude to psychiatry was partly related to the discipline itself, psychiatrists, or psychiatric patients. Female gender and previous experience with psychiatry, particularly curriculum-related and personal experience, were important predictors of all outcomes. Students in the first years of medical training were more interested in pursuing psychiatry as a career. Furthermore, the country of the medical school was related to the outcomes. However, statistical models explained only a small proportion of variance. The findings indicate that particularly curriculum-related experience is important for determining attitudes toward psychiatry, interest in the subject and self-predicted professional career choice. We therefore encourage the provision of opportunities for clinical experience by psychiatrists. However, further predictor variables need to be considered in future studies.

## Introduction

For two decades, there has been growing worldwide concern that psychiatry as a profession, its identity, and image is in crisis (1–4). Moreover, there is an imbalance between the high numbers affected by mental disorders (5–7), the high and increasing worldwide burden attributable to mental disorders in terms of disability-adjusted life years (DALYs), years of life lost to premature mortality (YLL) and years lived with disability (YLD) (8), and the declining numbers of young academics choosing psychiatry as a career (4, 9–13). Despite its achievements, psychiatry faces challenges that might contribute to the dearth of psychiatrists and to resource shortage in the field: namely, the questionable validity of diagnostic definitions, doubts about the effectiveness of therapeutic interventions due to negative or contradictory findings, and the methodological quality of related research, opposing ideologies such as biological psychiatry vs. psychotherapy, the resulting unclear role profile of the psychiatrist, patient criticism of the formerly paternalistic discipline, overlapping responsibilities of psychiatrists and other professions (e.g., neurologists, psychologists), and, finally, its low status within medicine and society (4). Accordingly, psychiatric organizations have launched initiatives to better understand the reluctance medical students appear to feel toward psychiatry and to improve the recruitment of young academics into the field (12, 14–17).

In order to learn more about medical students’ views of psychiatry, numerous studies have focused on their attitudes toward psychiatry and their intended or definite career choice in order to obtain a deeper insight into the factors which might contribute to a better standing (18–29). Most studies indicate a discrepancy between positive attitudes toward psychiatry and low willingness to work in the field (18, 21, 22). However, attitude toward psychiatry, interest in psychiatry, and intended career choice are positively interrelated (18, 20, 21). In particular, medical students acknowledge the value of psychotherapy (21), view psychiatry as intellectually challenging and personally rewarding, but are reported to entertain a certain skepticism toward factors such as scientific standing, status, prestige and financial prospects, psychiatrists, patients, and treatment (18). In previous studies, female medical students and those with previous experience with psychiatry had consistently more positive attitudes (18, 20, 21, 23, 24, 28, 29), higher general interest in psychiatry (18), or higher interest in psychiatry as a career (20, 22, 25–28). Attitudes toward psychiatry and the definite career choice might also depend on characteristics of the medical school concerned, such as selection of students by non-teachable competencies, cultural diversity among medical students or the medical school’s focus on psychiatric education, and the supportiveness of the faculty (18, 20). Furthermore, how the number of study years affects attitudes (18, 24), interest, or career choice remains unclear.

However, previous research also reveals limitations. It does not indicate the most important predictors of attitudes toward psychiatry, interest in psychiatry, or intended career choice because most studies are just descriptive and did not use multivariable modeling (21–24, 26, 27, 29). In particular, it remains unclear as to which type of previous experience with psychiatry (e.g., clinical experience in the curriculum, private contact with people with mental illness or work-related experience) is mainly associated with medical students’ perceptions of psychiatry. Samples are frequently small to moderately sized (*N* ≤ 500) (21–26, 28, 29). Finally, there is usually no hierarchical modeling in cases of multiple catchment areas (22, 26, 27), although the generalizability of results across settings is assumed without confirmatory evidence.

Accordingly, this study aimed to contribute to a better understanding of predictors of medical students’ current perceptions and attempted to avoid the limitations of previous research. Therefore, we multivariably and hierarchically analyzed sociodemographic and education-specific predictor variables such as gender and diverse types of previous experience with psychiatry (see below) for three outcomes: (1) attitudes toward psychiatry, (2) interest in psychiatry, and (3) estimated likelihood of working in psychiatry. To achieve a sufficient sample size, we used an innovative recruitment approach *via* social media. We further considered multiple medical schools with a German language curriculum: namely, in Germany, Switzerland, Austria, and Hungary. There are, of course, differences between these countries as well, which include lower thresholds in selecting prospective medical students and lower tuition fees in Hungary, the highest density of psychiatrists in Switzerland, followed by Germany and Hungary (30); no officially approved and independent mental health policy or program in Hungary (11, 31).

## Materials and Methods

### Data Collection

Data were collected electronically by a secure web application called “Research Electronic Database Capture (REDCap)” for constructing and managing online surveys and databases (32, 33). The link was distributed on Facebook *via* message boards and student associations all over medical schools with a German language curriculum (or program) across Germany, Switzerland, Austria, and Hungary (34). Accordingly, we used snowball sampling based on person-to-person subject recruitment (35): “Snowball sampling yields a study sample through referrals made among people with important characteristics for the research question. It is particularly applicable when the focus of study is on a sensitive issue, and thus requires the knowledge of insiders to locate people for study.”

Data collection started on November 24, 2016 and ended on January 10, 2017.

The Cantonal Ethics Committee Bern (Kantonale Ethikkomission Bern, KEK-BE), responsible for the study site, filed a letter of non-competence and stated no objection. Accordingly, approval by the KEK-BE was not required because the study type was exempt from the local legislation on research involving human beings (Human Research Act, Article 2, Section 1).

The participants declared their informed consent by participating in the study after having been fully informed about its content. They themselves decided to submit the manuscript anonymously or not. Those who gave their mailing address could participate in a lottery with the possibility to gain a smartphone by randomized selection at the end of recruitment. Data were stored and managed according to the data protection guidelines of the University of Bern (36, 37).

### Online-Questionnaire

The online questionnaire was composed of a German version (21) of the original Attitudes Toward Psychiatry (ATP-30) Scale (38). The frequently used ATP-30 measures medical students’ attitudes toward psychiatry (21–24, 28) and has proven validity and reliability, as assessed in a Canadian sample of medical students (38). According to the author, the split-half reliability by Spearman–Brown was *r* = 0.90 in medical and *r* = 0.89 in O.T. students. The test stability varied depending on the length and type of the test–retest period between *r* = 0.51 and *r* = 0.87, with a value of *r* = 0.69/*r* = 0.67 before and after psychiatry-related experience in second-, third-, and fourth-year medical students and O.T. students.

The German ATP-30-G showed high internal consistency (21). It comprises 30 Likert-scale items, with 5 = strongly disagree and 1 = strongly agree. The questionnaire includes diverse statements related to mental illness and psychiatric patients, psychiatric treatment and institutions, psychiatrists, education, knowledge, and psychiatry as a career choice. To calculate a sum score, scores of positively phrased items were subtracted from 6, resulting in higher values for a more positive attitude, and finally, item scores of positively and negatively phrased items were summed up. The sum score ranged between 30 and 150 points. A sum score of 90 would represent “the logical neutral point of the scale” (38).

We also asked participants about their general interest in the discipline of psychiatry and how likely it seemed to them that they would choose psychiatry as a career (21). Responses were given on a visual analog scale ranging from 0 to 100. Although the ATP-30 also includes specific items on interest (e.g., “Psychiatry is unappealing because it makes so little use of medical training”; “It is interesting to unravel the cause of a psychiatric illness”; “Psychiatric patients are often more interesting to work with than other patients.”) or career preference (e.g., “I would like to be a psychiatrist”), we preferred the above-mentioned global and probably more robust outcome measures. However, we conducted statistical analyses for both outcome variables on the professional preference as measured by the visual analog scale and the respective ATP item.

Predictor variables included the following: age (transformed by centering on the mean of 24 years), gender (male vs. female), nationality (German, Swiss, Austrian, Hungarian/other), medical school and country of medical school (German, Swiss, Austrian, Hungarian), study year (1st- to 4th semester [first 2 years, preclinical study period], 5th- to 10th semester [third, fourth, and fifth year, clinical part], and >10 semesters [beginning of clinical rotations]), and previous experience with psychiatry and type of experience (curriculum-related, personal, work-related, other). Curriculum-related experience included practical activities such as clinical traineeship/academic assistance. Further possible free answers under “other experience” related to training or internships and teaching, usually referring to medical training and only rarely to premedical education were grouped in this category. Personal experience included experiences with psychiatry related to oneself (e.g., psychotherapy) or to one’s personal environment, e.g., having friends or family members with a mental disorder. We also grouped possible free answers into this category if they were related to having mental health care professionals as parents or previous voluntary work in the social sector. Work-related experience referred to medical paid work during the course of medical studies (e.g., night shift on a ward). We also grouped information on professional work due to previous training (e.g., as a nurse or physiotherapist) into this category.

### Sample Selection

**Figure 1:**
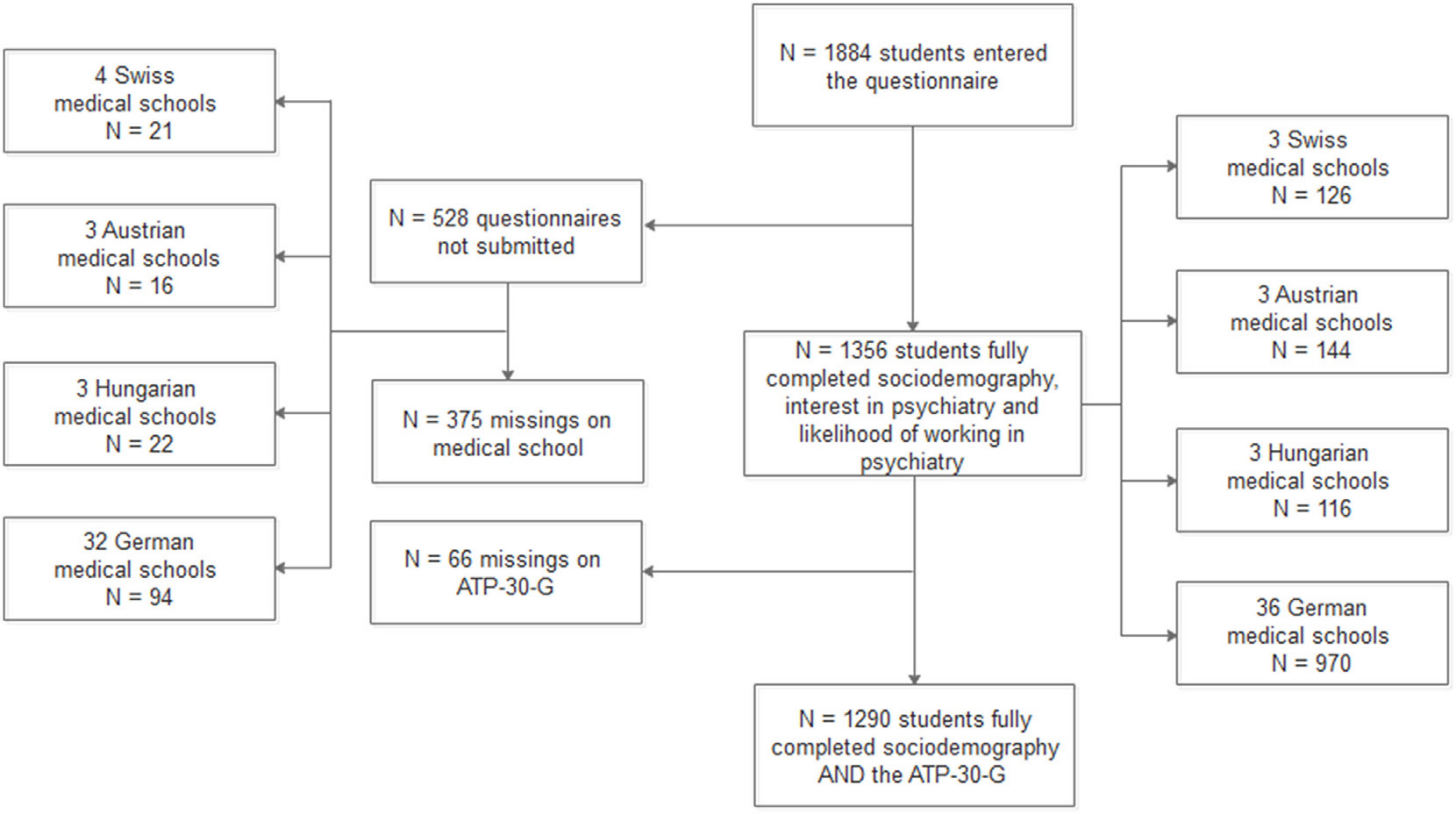
Sample selection procedure.

The sample selection procedure is illustrated in Figure 1. Of 1,884 students, 1,356 (72%) students from 45 medical schools submitted the online questionnaire and gave full information on predictor variables, interest in psychiatry, and likelihood of working in psychiatry. A subsample of 1,290 students gave full information on the ATP-30-G.

### Statistical Analyses

To avoid data loss, we used both samples described above (*N* = 1,356 and 1,290) for statistical analyses on outcomes.

First, we described the sample and compared sample characteristics across outcomes by *t*-test, ANOVA and Pearson’s correlation. We further analyzed the relationship between outcomes by Pearson’s correlation.

Second, we calculated linear mixed models (LMMs) (39, 40) by using the ATP-30-G sum score, interest in psychiatry, and estimated likelihood of working in psychiatry as outcome variables. LMMs are eligible for continuous outcome variables with normally distributed residuals, which may, however, not be independent or have constant variance (40). The independent variables may involve fixed effects with unknown constant parameters and random effects with levels of factors regarded as having been randomly sampled from a larger population (40). Using traditional regression models in nested data might lead to Type-I error inflation (39), whereas mixed models effectively address this issue (41). We considered the group variable “medical school” as a random variable at level 1, and other predictor variables as fixed effects. Interaction effects of the mixed-effects models were visualized by graphing marginal means.

Third, to evaluate the robustness of the mixed-model findings, we calculated complementary models by robust regression, using the Huber M estimation method,^1^ and quantile (median) regression.^2^ These methods are suitable for data contaminated with outliers and heterogeneous conditional distributions, and therefore appropriate for the outcome variables “interest in psychiatry” and “likelihood of working in psychiatry.” Robust regression indicates the amount of variance explained by *R*^2^ in order to assess the relative predictive capability of the statistical models. Results of all three regression types were illustrated by coefficient plots for each outcome.

Fourth, we considered the item “I would like to be a psychiatrist” as measured by the ATP-30-G as an outcome variable, with a higher score indicating a more positive attitude. We calculated a General Linear Model with cumulative logit function^3^ and the random-effect “medical school.”

Statistical models were run in SAS/STAT, Version 9.4 (42) using the procedures “proc mixed,” “proc robustreg” and “proc quantreg,” and “proc glimmix.” The plot of margins was generated in SPSS, version 23 for Windows (43). Coefficient plots were generated by Stata, version 14.2 for Mac (44).

## Results

### Non-Response and Sample Selection

A comparison of students who submitted the questionnaire (*N* = 1,356 responders) with those who did not but who entered sociodemographic information (*N* = 158 non-responders) revealed no statistically significant differences in age, gender, nationality, and number of semesters (Table S1A in Supplementary Material). The samples differed with respect to the country of the medical school, with more responders (71.5%) than non-responders (61.4%) enrolled in Germany and more responders (36.1%) than non-responders (23.2%) mentioning previous psychiatric experience. The two groups did not differ in “interest in psychiatry” (*N* = 11 missings in non-responders) and “likelihood of working in psychiatry” (*N* = 7 missings in non-responders), whereas findings on “attitudes toward psychiatry” were not interpretable (*N* = 145 missings in non-responders).

A comparison of the students who gave their mailing address and therefore took part in the lottery (*N* = 1,163) with those who did not (*N* = 193) showed that they differed statistically only with respect to age and gender, with women preferring to remain anonymous (Table S1B in Supplementary Material). The two groups differed neither in other sociodemographic or education-specific variables nor concerning attitude, interest or professional preference scores.

### Sample Description

Tables 1 and 2 present basic sample characteristics.

**Table 1:** Sample characteristics.

		Sample: *N* = 1,290^a^		Sample: *N* = 1,356^b^	
Sample characteristics		*N*	(%)	*N*	(%)
Age (mean, SD), years		24	(3.79)	24	(3.74)

Gender					
	Female	903	(70.0)	955	(70.4)
	Male	387	(30.0)	401	(29.6)

Nationality					
	Swiss	110	(8.5)	117	(8.6)
	Austrian	102	(7.9)	111	(8.2)
	Hungarian/other	58	(4.5)	63	(4.6)
	German	1,020	(79.1)	1,065	(78.5)

Medical school, country					
	Swiss	118	(9.1)	126	(9.3)
	Austrian	135	(10.5)	144	(10.6)
	Hungarian	111	(8.6)	116	(8.6)
	German	926	(71.8)	970	(71.5)

Semesters					
	1st–4th semester	245	(19.0)	256	(18.9)
	5th–10th semester	771	(59.8)	810	(59.7)
	>10th semester	274	(21.2)	290	(21.4)

Previous experience					
	Yes	458	(35.5)	489	(36.1)
	No	832	(64.5)	867	(63.9)

Type of previous experience					
	Curriculum-related	211	(16.4)	222	(16.4)
	Personal	155	(12.0)	169	(12.5)
	Work-related	92	(7.1)	98	(7.2)
	No experience	832	(64.5)	867	(63.9)

*^a^Complete information on the German version of the Attitudes Toward Psychiatry Scale*.

*^b^Full sample*.

**Table 2:** Mean outcome scores by sample characteristics.

		ATP-30-G sum score*N* = 1,290		Interest in psychiatry*N* = 1,356		Likelihood of working in psychiatry*N* = 1,356	

		Mean	SD	Mean	SD	Mean	SD
**Sample characteristics**							
Age (mean, SD), years							
Gender							
	Female	112.4	12.9	65.4	24.4	37.1	28.4
	Male	109.2	14.2	58.8	25.8	30.8	28.0

Nationality							
	Swiss	112.2	13.5	63.8	23.4	35.2	25.2
	Austrian	114.3	12.1	59.8	27.3	31.1	25.7
	Hungarian/other	108.7	14.6	57.9	30.8	37.0	32.1
	German	111.2	13.4	64.1	24.5	35.6	28.8

Medical school, country							
	Swiss	111.9	13.3	64.6	23.1	34.9	24.9
	Austrian	112.2	30.6	58.4	26.8	30.6	25.6
	Hungarian	106.4	13.7	57.4	24.5	28.0	27.1
	German	111.8	13.3	64.8	24.9	36.8	29.2

Semesters							
	1st–4th semester	108.8	13.4	63.6	23.7	37.1	26.8
	5th–10th semester	111.5	13.3	63.2	25.0	34.1	27.5
	>10th semester	113.5	13.3	63.9	26.1	36.7	31.9

Previous experience							
	Yes	115.7	13.1	73.0	23.0	46.2	30.6
	No	109.1	12.9	58.1	24.5	29.1	25.1

Type of previous experience							
	Curriculum-related	117.2	13.0	73.7	23.5	48.7	31.2
	Personal	115.7	12.9	75.9	20.1	46.1	29.1
	Work-related	112.2	13.2	66.2	25.5	40.6	31.4
	No experience	109.1	12.9	58.1	24.5	29.1	25.1

*An ANOVA was performed in the case of more than two categories within variables; a t-test was done in the case of two categories within variables; a Pearson’s correlation was done in the case of a continuous variable*.

ATP-30-G sum score: ****P < 0.0001: age, gender, semesters, previous experience, type of previous experience, **P < 0.01: medical school, country, n.s.: nationality.

Interest in psychiatry: ****P < 0.0001: gender, previous experience, type of previous experience, **P < 0.01: medical school, country, n.s.: age, nationality, semesters.

Likelihood of working in psychiatry: ****P < 0.0001: gender, previous experience, type of previous experience, **P < 0.01: medical school, country, *P < 0.05: age, n.s.: nationality, semesters.

The majority were females and German students enrolled at a medical school in Germany, in the clinical part of their studies (5th–10th semester) and without previous experience in psychiatry. Most previous experience was curriculum-related.

Females and those with previous experience—especially curriculum-related or personal experience—had higher scores in attitudes, interest in psychiatry, and estimated likelihood of working in psychiatry. Students from a medical school in Hungary had lower scores in all outcomes. Furthermore, a shorter length of medical study was associated with lower attitude scores (Table 2).

### Relationship between Outcome Variables

The outcome variables were strongly and positively correlated. The ATP-30-G sum score correlated with “interest in psychiatry” by *r* = 0.59 (*p* < 0.0001) and with “likelihood of working in psychiatry” by *r* = 0.54 (*p* < 0.0001). Interest in psychiatry and likelihood of working in psychiatry correlated by *r* = 0.66 (*p* < 0.0001). Likelihood of working in psychiatry and the ATP-30-G-item “I would like to be a psychiatrist” correlated by *r* = 0.79 (*p* < 0.0001; due to missing values in the ATP item *N* = 1,353).

### Outcome Scores

On average, medical students had a positive attitude toward psychiatry. The mean ATP-30-G sum score was 111.42 (SD = 13.35). The mean “interest in psychiatry score” was 63.44 (SD = 25.01), and the mean “likelihood of working in psychiatry score” was 35.22 (SD = 28.39).

**Figure 2:**
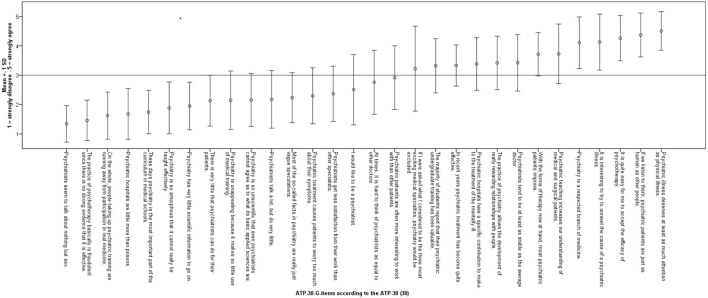
Mean and SD of single German version of the Attitudes Toward Psychiatry Scale (ATP-30-G) items.

Figure 2 shows means and SD for each ATP-30-G item according to the ATP-30. For readability, we inversely coded all items, with 5 indicating “strongly agree” and 1 indicating “strongly disagree.” The results show that the medical students surveyed acknowledged psychiatry as a medical discipline based on scientific evidence. They believed in the efficacy of psychotherapy and, to a lesser extent, in psychiatric institutions. Attitudes toward psychiatrists were positive to ambiguous. They answered neutrally or negatively on items indicating a professional preference toward psychiatry. With respect to education, they fluctuated between a negative and a positive attitude. Overall, medical students had a neutral to positive attitude toward mental disorders and psychiatric patients.

### Linear Mixed and Complementary Models

**Figure 3:**
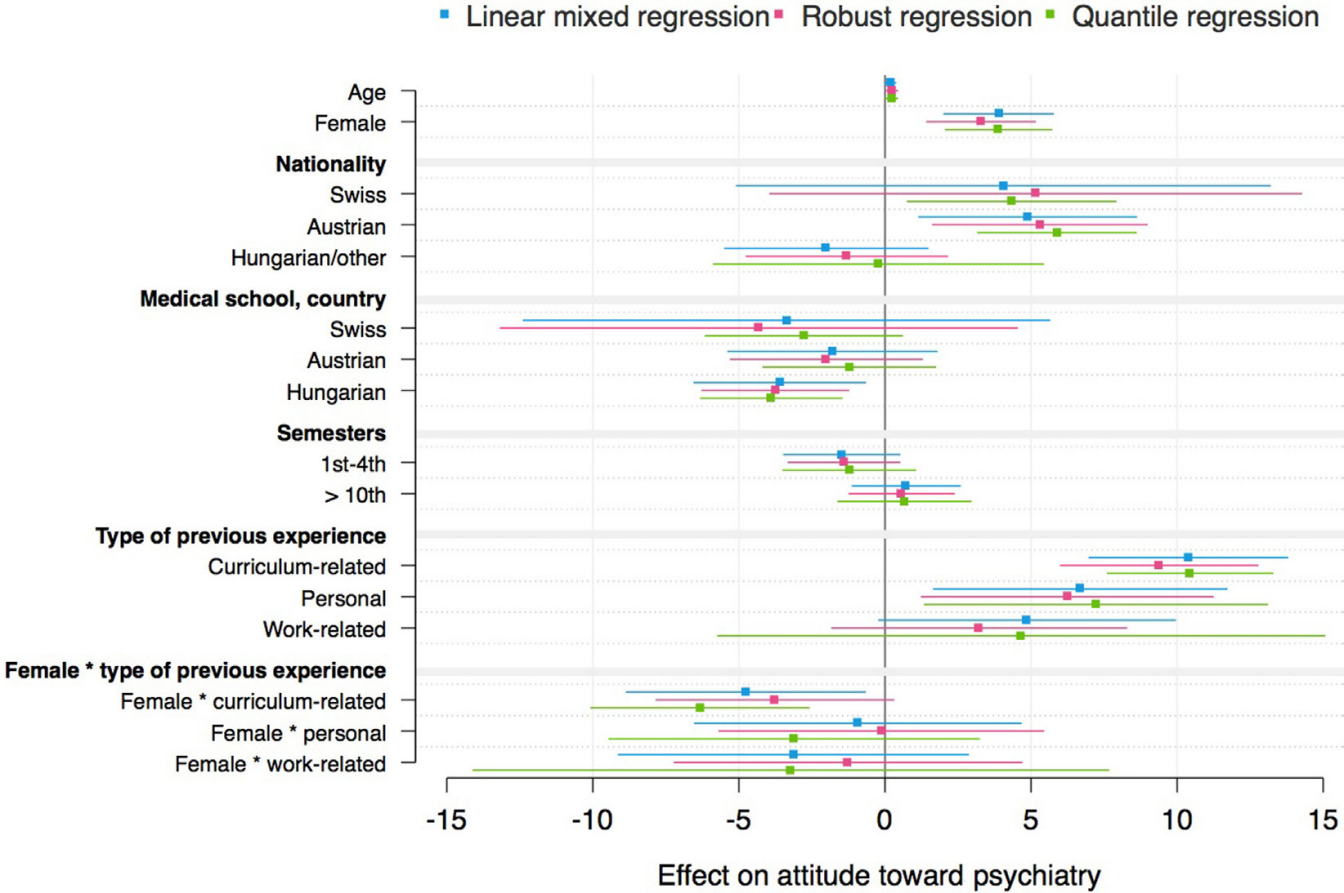
Coefficient plot: German version of the Attitudes Toward Psychiatry Scale sum score (robust regression: *R*^2^ = 0.086).

**Figure 4:**
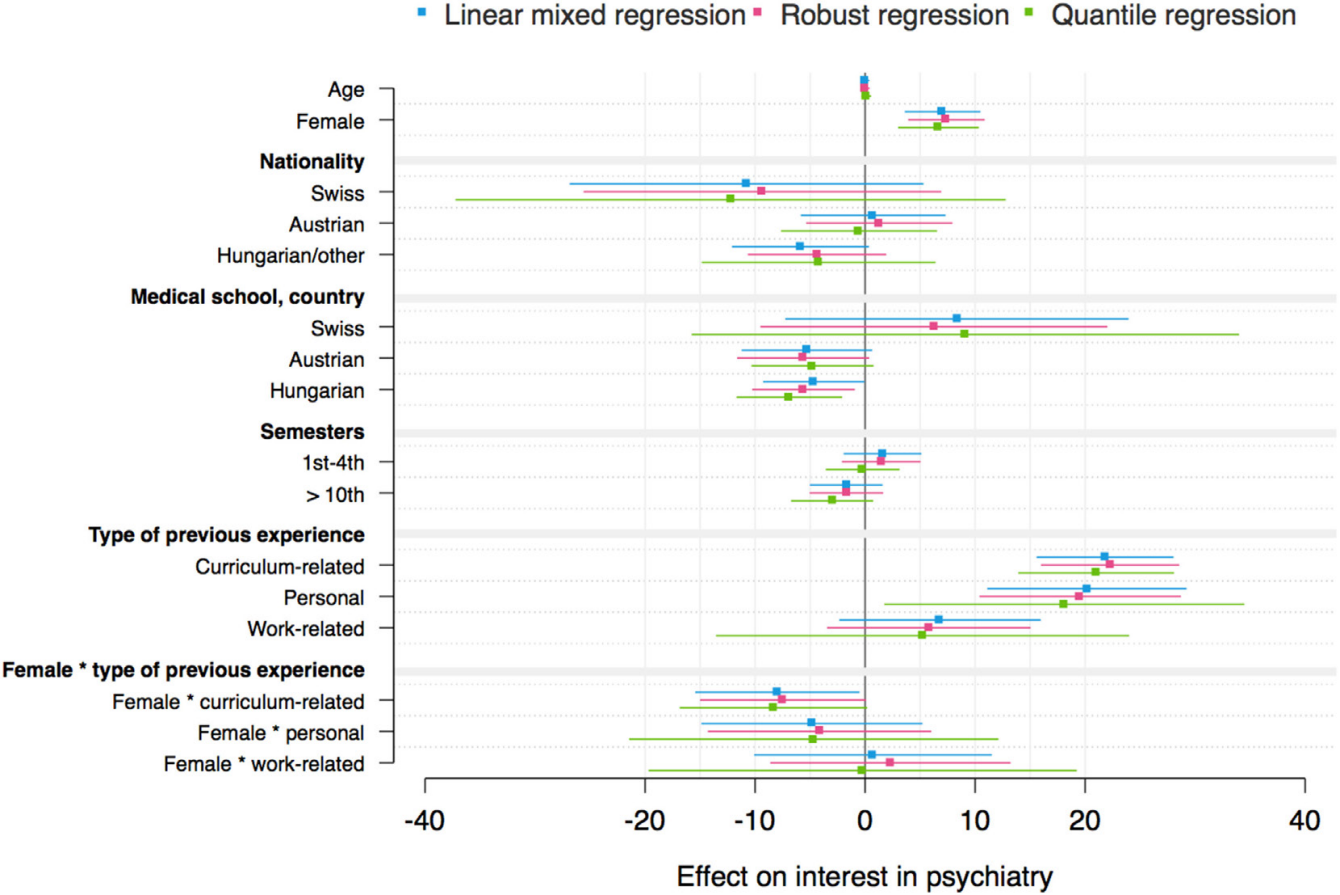
Coefficient plot: interest in psychiatry (robust regression: *R*^2^ = 0.106).

**Figure 5:**
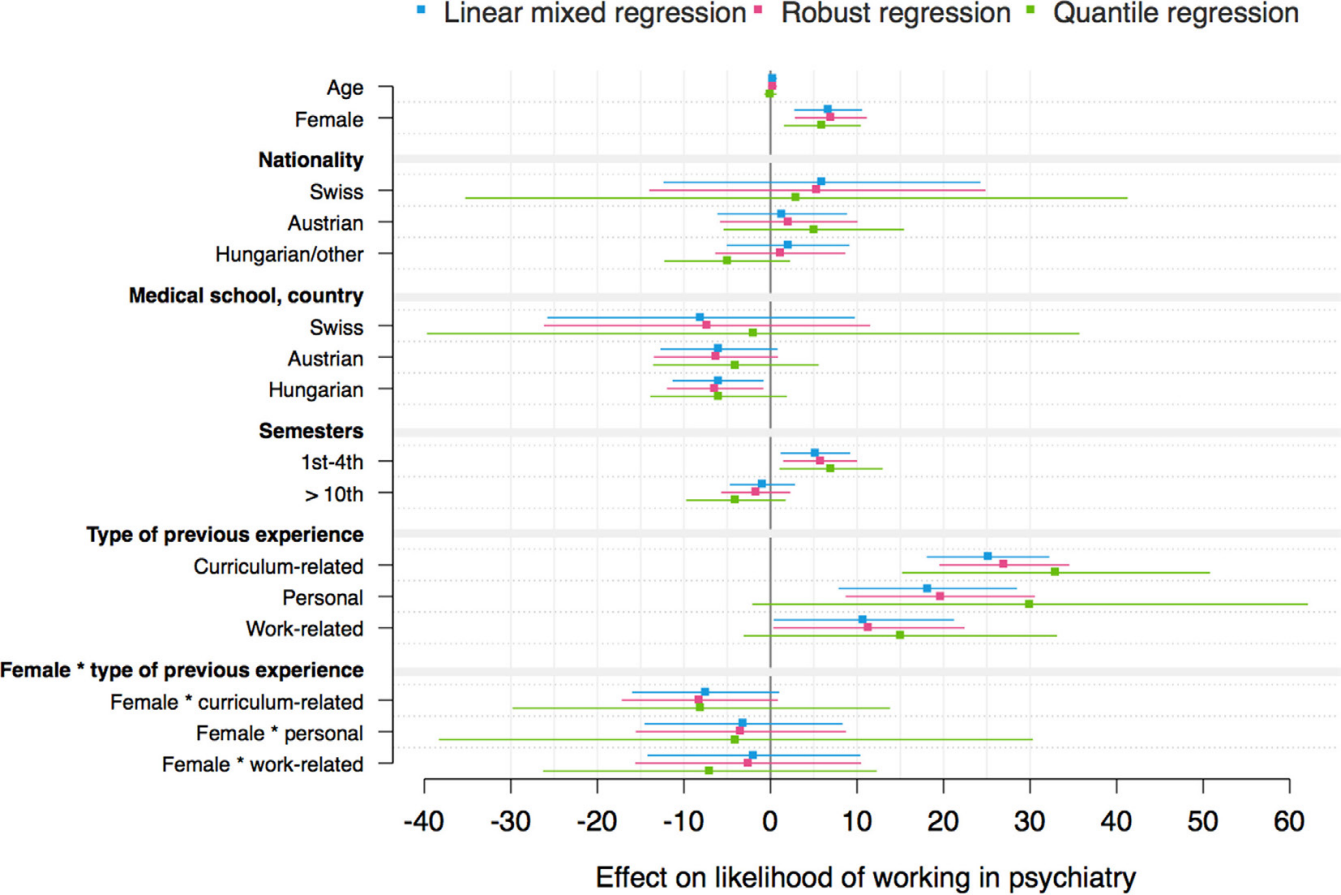
Coefficient plot: likelihood of working in psychiatry (robust regression: *R*^2^ = 0.105)

The mixed models for the three outcome variables “attitudes toward psychiatry,” “interest in psychiatry,” and “likelihood of working in psychiatry” and the interaction effect between gender and type of previous experience are shown in Tables 3–5. Mixed models including the interaction effect between gender and previous experience as a dichotomous variable are demonstrated in Tables S2–S4 in Supplementary Material. Complementary models are shown by coefficient plots (Figures 3–5). An additional model on the ATP item “I would like to be a psychiatrist” is shown in Table S5 in Supplementary Material.

**Table 3:** Mixed-effects models: German version of the Attitudes Toward Psychiatry Scale sum score (*N* = 1,290); interaction effect gender × type of previous experience.

Sample characteristics		Estimate	SE	df	*t*-Value/*z*-value	Pr > |*t*|
Intercept		106.85	0.92	347	116.43	<0.0001
Age (centered), years		0.18	0.11	1,290	1.69	0.091

Gender						
	Female	**3.89**	**0.96**	**1,286**	**4.03**	**<0.0001**
	Male (Ref)^a^					

Nationality						
	Swiss	4.05	4.67	1,258	0.87	0.386
	Austrian	**4.89**	**1.91**	**1,263**	**2.56**	**0.011**
	Hungarian/other	−2.01	1.78	1,289	−1.13	0.261
	German (Ref)^a^					

Medical school, country						
	Swiss	−3.37	4.61	905	−0.73	0.465
	Austrian	−1.80	1.84	60.2	−0.98	0.332
	Hungarian	**−3.60**	**1.51**	**21.9**	**−2.39**	**0.026**
	German (Ref)^a^					

Semesters						
	1st–4th semester	−1.48	1.02	636	−1.44	0.149
	>10th semester	0.73	0.95	921	0.76	0.445
	5th–10th semester (Ref)^a^					

Type of previous experience						
	Curriculum-related	**10.38**	**1.74**	**1,284**	**5.96**	**<0.0001**
	Personal	**6.69**	**2.57**	**1,267**	**2.57**	**0.009**
	Work-related	4.87	2.60	1,284	1.87	0.062
	No experience (Ref)^a^					

Gender × type of previous experience						
	Female × curriculum-related	**−4.76**	**2.10**	**1,273**	**−2.27**	**0.023**
	Female × personal	−0.93	2.86	1,272	−0.33	0.745
	Female × work-related	−3.14	3.06	1,284	−1.02	0.306

Random effects						
Residual		159.35	6.40		24.89	<0.0001
Intercept		1.30	1.64		0.80	0.213

*^a^Ref, reference category; fit statistics: AIC = 10,249.8 and BIC = 10,284.1*.

*Statistically significant findings (*p* < 0.05) are shown in bold font*.

**Table 4:** Mixed-effects models: interest in psychiatry (*N* = 1,356); interaction effect gender × type of previous experience.

Sample characteristics		Estimate	SE	df	*t*-Value/*z*-value	Pr > |*t*|
Intercept		54.64	1.62	1,356	33.66	<0.0001
Age (centered), years		0.01	0.20	1,356	0.03	0.974

Gender						
	Female	**7.05**	**1.76**	**1,356**	**4.01**	**<0.0001**
	Male (Ref)^a^					

Nationality						
	Swiss	−10.77	8.21	1,356	−1.31	0.189
	Austrian	0.73	3.36	1,356	0.22	0.827
	Hungarian/other	−5.88	3.18	1,356	−1.85	0.064
	German (Ref)^a^					

Medical school, country						
						
	Swiss	8.35	7.96	1,356	1.05	0.294
	Austrian	−5.29	3.03	1,356	−1.75	0.081
	Hungarian	−**4.66**	**2.36**	**1,356**	−**1.98**	**0.048**
	German (Ref)^a^					

Semesters						
	1st–4th semester	1.58	1.80	1,356	0.88	0.381
	>10th semester	−1.71	1.69	1,356	−1.01	0.311
	5th–10th semester (Ref)^a^					

Type of previous experience						
	Curriculum-related	**21.81**	**3.17**	**1,356**	**6.87**	**<0.0001**
	Personal	**20.16**	**4.62**	**1,356**	**4.36**	**<0.0001**
	Work-related	6.81	4.67	1,356	1.46	0.145
	No experience (Ref)^a^					

Gender × type of previous experience						
	Female × curriculum-related	−**7.97**	**3.81**	**1,356**	−**2.09**	**0.037**
	Female × personal	−4.83	5.13	1,356	−0.94	0.346
	Female × work-related	0.72	5.51	1,356	0.13	0.897

Random effects						
Residual		553.65	21.26		26.04	<0.0001
Intercept		–	–	–	–	–

*^a^Ref, reference category; fit statistics: AIC = 12,449.4 and BIC = 12,481.9*.

*Statistically significant findings (*p* < 0.05) are shown in bold font*.

**Table 5:** Mixed-effects models: likelihood of working in psychiatry (*N* = 1,356); interaction effect gender × type of previous experience.

Sample characteristics		Estimate	SE	df	*t*-Value/*z*-value	Pr > |*t*|
Intercept		24.93	1.85	1,356	13.49	<0.0001
Age (centered), years		0.26	0.23	1,356	1.17	0.241

Gender						
	Female	**6.66**	**2.00**	**1,356**	**3.33**	**0.001**
	Male (Ref)^a^					

Nationality						
	Swiss	5.94	9.34	1,356	0.64	0.525
	Austrian	1.35	3.82	1,356	0.35	0.724
	Hungarian/other	2.04	3.62	1,356	0.56	0.573
	German (Ref)^a^					

Medical school, country						
						
	Swiss	−8.02	9.06	1,356	−0.88	0.377
	Austrian	−5.95	3.45	1,356	−1.72	0.085
	Hungarian	−**6.06**	**2.68**	**1,356**	−**2.26**	**0.024**
	German (Ref)^a^					

Semesters						
	1st–4th semester	**5.19**	**2.05**	**1,356**	**2.52**	**0.012**
	>10th semester	−0.92	1.92	1,356	−0.48	0.631
	5th–10th semester (Ref)^a^					

Type of previous experience						
	Curriculum-related	**25.14**	**3.61**	**1,356**	**6.96**	**<0.0001**
	Personal	**18.17**	**5.26**	**1,356**	**3.45**	**0.001**
	Work-related	**10.79**	**5.32**	**1,356**	**2.03**	**0.043**
	No experience (Ref)^a^					

Gender × type of previous experience						
	Female × curriculum-related	−7.49	4.34	1,356	−1.73	0.084
	Female × personal	−3.11	5.84	1,356	−0.53	0.595
	Female × work-related	−1.91	6.28	1,356	−0.30	0.761

Random effects						
Residual		717.79	27.57		26.04	<0.0001
Intercept		–	–	–	–	–

*^a^Ref, reference category; fit statistics: AIC = 12,801.5 and BIC = 12,834.0*.

*Statistically significant findings (*p* < 0.05) are shown in bold font*.

In general, the variance between medical schools was not statistically significant in any mixed model. However, the residual variance revealed that there were individual differences among students within medical schools. The robust regression models revealed *R*^2^ values between 8 and 11%.

**Figure 6:**
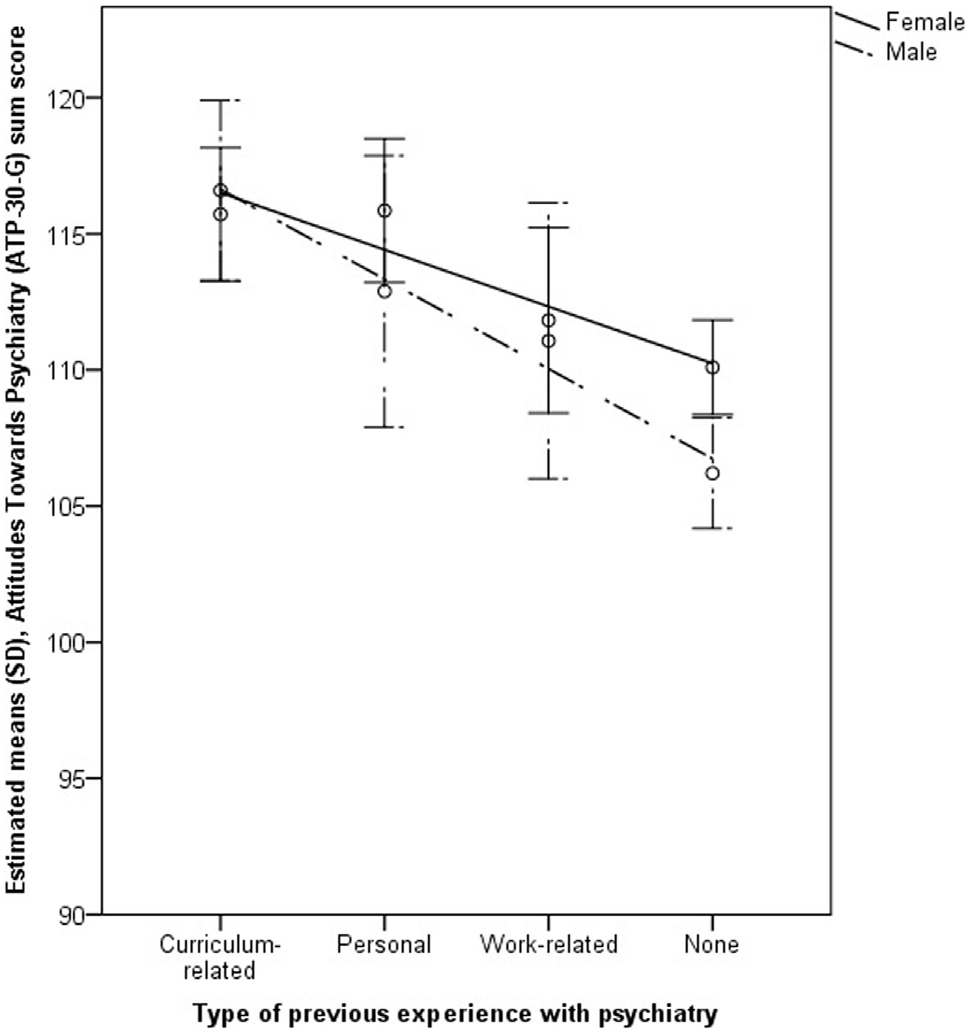
Margins plot: gender by type of experience controlled for other predictors: German version of the Attitudes Toward Psychiatry Scale (ATP-30-G) sum score.

Being female and having previous experience with psychiatry (compared with no experience), especially curriculum-related and personal experience, were associated with higher attitude toward, interest in and likelihood of working in psychiatry scores/ATP-30-G-item score “I would like to be a psychiatrist” (Tables 3–5; Figures 3–5; Tables S2–S5 in Supplementary Material). Furthermore, there was an interaction effect between females and previous experience with psychiatry only with respect to attitudes, as shown in Table 3. Notably, the ATP-30-G sum score did not differ in men and women with experience (estimated mean, men = 114.63 [SE = 1.37]; estimated mean women = 115.06 [SE = 0.98]), but men without experience had lower attitude scores than did women (estimated mean, men = 106.23 [SE = 1.04]; estimated mean women = 110.10 [SE = 0.89]). In particular, curriculum-related experience was linked with comparable ATP-30-G sum scores for both genders (Figure 6). However, the interaction effects did not hold for robust regression (Figure 3).

Furthermore, the study year, nationality, and medical school were related to the outcome scores. Austrian nationality was statistically consistently linked with higher attitude scores than German nationality (Table 3; Table S2 in Supplementary Material; Figure 3).

Being enrolled at a Hungarian medical school was consistently related to lower attitude scores (Table 3; Table S2 in Supplementary Material; Figure 3), and partly to lower interest (Table 4; Figure 4) or lower likelihood of working in psychiatry (Table 5; Table S4 in Supplementary Material; Figure 5) than being enrolled at a German medical school.

Students in the preclinical part of their studies (i.e., 1st–4th semester) showed a higher estimated likelihood of working in psychiatry/higher ATP-30-G-item score “I would like to be a psychiatrist” compared with students in the clinical part (5th–10th semester) (Table 5; Tables S4 and S5 in Supplementary Material; Figure 5).

## Discussion

Considering the lack of young academics entering the field of psychiatry, this study aimed at analyzing medical students’ perceptions of the discipline and related predictors (e.g., gender and previous experience). We also attempted to avoid certain limitations of previous research, such as mostly descriptive studies and limited sample size. We found high interest in, but low professional preference for, psychiatry and an overall positive to ambiguous attitude toward psychiatry. Multivariable analyses revealed that gender and experience with psychiatry, particularly curriculum-related and personal experience, were among the statistically significant predictors. However, the statistical models explained at most 11% of the variance.

By and large, our findings on attitudes agree with previous research (18, 21, 22). Overall, it seems that attitudes toward psychiatry have not changed over the last 20 years (21)—a time period in which new teaching models have arisen and knowledge about mental disorders has increased. Like others, we identified positive attitudes toward psychotherapy (21). According to some (21) but not all previous findings (18, 21, 45), we also found positive attitudes toward people with mental disorders. However, as previously shown, attitudes toward psychiatrists were ambiguous (18, 21) and the preference for psychiatry over other medical disciplines remained unclear (21, 25).

Women had more positive attitudes toward psychiatry, higher interest in psychiatry and a higher estimated likelihood of working in psychiatry in our study. This corresponds to most previous results (18, 20, 22–24, 28, 29) but not to all (21, 25, 27). The finding is also congruent with the fact that more women later seek a specialization in psychiatry (20), which might be linked with lifestyle aspects in terms of flexible working hours (18, 20, 25) and work-life-balance (21, 25), or with the social aspect of psychiatry rather than with medical-technical aspects (46). According to our results and previous evidence (23), attitudes toward psychiatry may improve in men after (curriculum-related) experience, whereas women’s attitudes are positive irrespective of previous experience.

The positive association between previous experience with psychiatry and attitudes (18, 21, 23, 24, 47), interest in psychiatry (18), or intended career choice (20, 22, 25–27) is in line with previous evidence. Others have also identified the importance of previous personal or work-related experience with psychiatry for (early) career choice (25, 27). Due to the cross-sectional design of our study, it is impossible to make inferences about causality, but studies with repeated measures have demonstrated that attitudes toward psychiatry (28, 29, 48) or a career interest (49) can improve after curriculum-related experience.

In the meantime, widely implemented methods combining theory and practice with concepts such as problem-based learning or bedside-teaching (50, 51) or other approaches (e.g., psychiatry electives, summer institutes, exchange programs, small group teaching, simulated patients, using movies, multidisciplinary seminars, integrated teaching, attitude questionnaires, and objective structured clinical examinations, etc.) could be promising in further improving attitudes toward psychiatry (23, 52), interest in psychiatry, and/or career choice (19, 26). In particular, scenario-based learning in different settings, such as primary care or general hospitals, could prepare medical students for a potential future in psychiatry (53, 54), even if findings on the setting of clinical experience with psychiatry are contradictory (26).

As findings on attitudes toward psychiatrists were positive to ambiguous, in particular concerning a neutral attitude toward the statement that psychiatrists are not thought of as being equal to other doctors, personal contact with psychiatrists through tutorials might be helpful. The professionals themselves could contribute to overcoming stigma and to obtaining an accurate picture of psychiatry (19), given that they are aware of self-stigma (55) and stigma against their own clientele (56). Furthermore, personal experience with psychiatry might be promoted by early volunteering opportunities to come in contact with the discipline. The effectiveness of any clinical experience might rely on factors such as personal contact with patients and the development of close relationships, seeing patients recover, perceived quality of the experience or improvement in clinical skills, and understanding patients’ feelings (48).

Due to our finding of a higher estimated likelihood of working in psychiatry in the first semesters, we, along with other authors (25), suggest that psychiatry as a medical discipline in a psychosocial context could be introduced earlier in the medical curriculum. However, it still remains unclear what effect an early introduction of psychiatry could have on students’ general attitudes toward psychiatry. In this respect, besides timing, teaching mental disorders as being based on multiple etiologies is important. Biological explanations of mental illness (57) might increase the acceptance of medical treatment, but a holistic understanding of the etiology of mental disorders could reduce social distance (58). In any case, according to medical students themselves, psychiatry should be better integrated into the general curriculum (54), which might refer to timing and content.

Furthermore, we found differences connected with the nationality of students and the country where medical schools were located. It remains unclear why medical students from Austria had higher attitude scores than German students, as both countries have a long tradition of psychiatric medicine. Hungarian medical schools were linked with lower attitude scores. This might be related to medical faculties, education, and/or limitations in mental health politics or working conditions (11). These differences illustrate the importance of the country of the medical school for analysing medical students’ attitudes toward psychiatry.

This study has strengths and limitations. We used an innovative recruitment approach in the form of snowball sampling on Facebook. Furthermore, we multivariably and hierarchically analyzed basic predictors of medical students’ perceptions of psychiatry by focusing on types of previous experience with psychiatry.

However, snowball sampling does not allow for inferences about representativity in any one place. Our data include a selection bias toward German medical students from German medical schools, which might reflect different country size but also might be due to the method of recruitment. Due to the nature of online questionnaires, we could not rule out invalid data entries; for instance, diverse items on career choice showed partly inconsistent findings.

A general positive attitude toward psychiatry and psychiatric patients or choosing psychiatry as a career does not rule out negative stereotypes toward people with mental disorders (56). Accordingly, it remains moot whether some ATP-30-G statements were rated based on social desirability. Furthermore, the phrasing of certain ATP-30 items could be viewed critically (e.g., “Psychiatrists seem to talk about nothing but sex.” or “If we listen to them, psychiatric patients are just as human as other people.”).

Our findings have implications for future research. More specific factors need to be addressed to account for a larger proportion of the variance in statistical models. Examples of this might be: components of medical training (e.g., lectures, special psychiatric modules or clinical placement and its quality, teacher–student relationships, supportiveness, and mentoring) (18, 20, 26), personal characteristics of medical students (e.g., personality factors) (20, 23), or family history and attitudes of close social networks (25). Furthermore, future studies should include multiple medical schools and their characteristics in hierarchical models in order to better understand students’ perceptions. In particular, comparable studies in other, non-German-speaking countries could help to better understand medical students’ views on psychiatry and their determining factors in different educational and health systems.

In summary, in an online survey of 1,356 medical students from medical schools with German language curricula in four European countries, female gender and previous experience, particularly curriculum-related and personal experience, determined attitudes toward and interest in psychiatry, and self-predicted career choice. Opportunities for clinical experience should be promoted in medical curricula, particularly for men. Further predictor variables might contribute to a larger proportion of explained variance in statistical models.

## Nomenclature

**Table:** 

ATP-30	Attitudes Toward Psychiatry Scale
ATP-30-G	German version of the Attitudes Toward Psychiatry Scale
REDCap	Research Electronic Data Capture
KEK-BE	Cantonal Ethics Committee Bern; Kantonale Ethikkomission Bern
LMMs	linear mixed models

## Ethics Statement

The Cantonal Ethics Committee Bern (Kantonale Ethikkomission Bern, KEK-BE), responsible for the study site, filed a letter of non-competence and stated no objection. Accordingly, approval by the KEK-BE was not required because the study type was exempt from the local legislation on research involving human beings (Human Research Act, Article 2, Section 1).

## Author Contributions

ML designed the study including the sample selection procedure and reviewed the manuscript. BS and his research group translated the English original version of the ATP-30 by Burra et al. (38). We further used the same predictor and “mediator variables” as described in the study of Strebel et al. (21). AG designed the statistical analysis plan and reviewed the statistical analyses as well as the manuscript. IW designed the study, conducted the statistical analyses, and wrote the manuscript. MB did the recruitment *via* Facebook and used a subset of the data for her thesis. RS, CC, BS, TT, WR, and NR reviewed the draft paper and contributed to the interpretation of the data. All authors approved the final manuscript.

## Conflict of Interest Statement

The authors declare that the research was conducted in the absence of any commercial or financial relationships that could be construed as a potential conflict of interest.

## Footnotes

^1^Chen C. *Robust Regression and Outlier Detection with the ROBUSTREG Procedure [Paper 265–27]*. Cary, NC: SAS Institute Inc.

^2^Chen C. *An Introduction to Quantile Regression and the QUANTREG Procedure [Paper 213–30]*. Cary, NC: SAS Institute Inc.

^3^Schabenberger O. *Introducing the GLIMMIX Procedure for Generalized Linear Mixed Models [Working Paper]*. Cary, NC: SAS Institute Inc.
